# The Reproductive and Anatomical Characteristics of the Invasive Nutria (*Myocastor coypus* M.) in a Central European Population

**DOI:** 10.3390/ani15243524

**Published:** 2025-12-07

**Authors:** Balázs Bócsi, Zsolt Biró, Krisztián Katona

**Affiliations:** 1 Department of Wildlife Biology and Management, Institute for Wildlife Management and Nature Conservation, Hungarian University of Agriculture and Life Sciences, Páter Károly u. 1., H-2100 Gödöllő, Hungary; katona.krisztian@uni-mate.hu; 2 National Laboratory for Health Security, Hungarian University of Agriculture and Life Sciences, Páter Károly u. 1., H-2100 Gödöllő, Hungary

**Keywords:** coypu, invasive alien species, *Myocastor coypus*, non-native, rodent, semi-aquatic, Slovakia

## Abstract

The nutria or coypu (*Myocastor coypus* M.) is endemic to South America. However, this species invaded Central Europe in the 19th century. The rapid spread of the invasive nutria across Central Europe triggered countless ecological conflicts with the local species. In the current research, we surveyed two populations in Slovakia and compared the reproductive performance of this species to its reproductive performance in other countries, where it is native. A total of 69 nutria were harvested from the wild in 2022–2024. The result of the postmortem analysis reveals no intersexual differences in the body weight and length. A visual inspection of the uterus among 25 female specimens confirmed that 16 (64%) were pregnant, including 3 individuals with the body size characteristics of young nutrias. The pregnancy rate was estimated at 90% (9 out of 10) in spring and 47% (7 out of 15) in autumn. The average number of embryos per individual was 6.94 ± 2.22 for all females, and it was 6.27 ± 2.05 in spring and 8.8 ± 1.64 in autumn, with no significant difference between seasons (*p* > 0.05). No clear relationship between the Body Mass Index (BMI) and the fertility of females, i.e., the number of embryos, was observed. Our results highlight the crucial need to raise public awareness about the invasive characteristics of the species and contribute to the establishment of an effective intervention techniques, including the establishment of the necessary legal framework for eradicating emerging populations across Central European habitats.

## Simple Summary

The nutria is a non-native invasive species in Europe that causes significant damage to native ecosystems throughout the continent. The reproductive performance of this species in newly established territories in Central Europe is poorly understood. This research investigates the reproductive performance of this animal in a population in Slovakia. A total of 69 carcasses were collected during hunting season from Dlhá nad Váhom (Vághosszúfalu) and Dolné Saliby (Alsószeli). The postmortem investigation of carcasses revealed that the species was reproductively active in both spring and summer, with an average of seven embryos per female. These findings highlight the need for an intensive control programme to contain the rapid spread of the species and minimise its damage to local ecosystems.

## 1. Introduction

The nutria or coypu (*Myocastor coypus* M.) is one of the most widespread non-native mammals in the world [1]. The species is endemic to the South American continent [2]. This non-native semi-aquatic animal is not only present in its original habitat, but introduced populations are present in North America, Europe, East Africa, the Middle East, northern Asia, and the Far East [2,3,4,5,6]. In Europe, nutria is included in the list of invasive alien species of the European Union [7] due to its rapid spread across the continent [8].

Several populations of nutria have been reported in Central Europe [9,10,11,12]. The nutria had first been introduced in Europe in the late 19th century [4,13,14,15]. For example, reports from the former Czechoslovakia (now Czechia and Slovakia), state that the coypu was introduced in 1925, and 5 state farms and 800 family farms kept and bred them. Similarly, the nutria took an important part in the fur industry in the former German Democratic Republic (East Germany) and Federal Republic of Germany (West Germany). The nutria was kept as the most important livestock in the 1980s in East Germany, due to its fur and meat production. Around the same time, Poland became the biggest producer country of nutria fur by producing nearly 3,000,000 nutria furs and exporting 2,000,000 in 1980 [16]. In Hungary, the nutria was also introduced in the 20th century as livestock for fur production, and several wild populations were established throughout the country [17].

Although the designation of the species as a pest is widespread in Central Europe, the management and legal status are different across countries. For example, in Slovakia, the species is considered huntable during the entire year and its legal status varies between different provinces of Germany, and in Hungary it is not a game species. In addition to the legal status, other factors may also complicate the effective management of nutria from certain areas. Based on social media, it appears that many people have a positive attitude towards the species (e.g., they like feeding them in urban environments), which can hinder their eradication, because the citizens do not want the nutrias to be removed.

The appearance of the nutria triggers countless new conflicts, due to the fact that this rodent is able to cause widespread damages to native populations. In addition, nutria is known to negatively impact water management systems, nature conservation and wildlife management areas, as well as causing significant damage to agriculture in several European countries [18]. The nutria is responsible for one of the highest estimated costs of invasive alien species in the world, with USD 19 billion of damage (e.g., the value of crop losses and damage repair) [19], and its economic costs have exponentially increased during recent years [20]. Nutria’s burrowing activity contributes to the siltation and riverbank instability, which notably leads to the decline of the local waterfowl population in some areas [21]. However, one of the most significant damages of the nutria is caused in agriculture. The nutria can disturb hydrologic dynamics of invaded habitats by demolishing draining structures in both direct and indirect ways, which can lead to an increased risk of flooding [22]. Furthermore, several researchers contributed information on the diseases of the nutria (e.g., tularemia, trichinella [23], leptospirosis [24], tuberculosis [25]) that can cause epizootics in wild animals, domestic livestock and in humans [24,26,27].

The nutria is a highly reproductive species, which can explain its successful expansion across the world. Nutrias breed non-seasonally, which means two–three litters in a year, and the litter size can reach up to twelve offsprings [13], and the average size of the litter is six [28].

This species is able to mate during the whole year, which makes it possible to more efficiently adapt to the changing climate. Earlier studies report three main mating periods, considering their habitats (beginning of summer, middle of autumn, and end of winter) [29,30]. In the case of the male specimens, sperm is similarly being produced during the whole year, while the females are polyestrous [30]. In a study at the Po River in Italy, out of the 80 trapped and later euthanized nutrias, 29 were females, and 18 (62%) were pregnant [31].

In rodents, the body condition is directly linked to reproductive performance [32,33,34,35] and the same pattern can be expected to play an important role [32,36] in the case of the nutria. While other studies report a strong correlation between the Body Mass Index, larger litter size, and better survival, to the best of our knowledge, this pattern has not been investigated among invasive populations across Central Europe [37,38,39]. Detailed research on body condition and reproductive performance of wild nutria is scarce. According to a Czech experiment, the growth of male nutrias was significantly higher than the female individuals; the differences were 12% at three months of age and grew to 24% at eight months of age [40]. However, these individuals were farmed nutrias, fed by humans. In Japan, another research [41] reported that out of 72 adult females examined, 60 (83.3%) were pregnant at the time of examination, and the mean litter size was 6.5 ± 2.4. Although they mentioned the method of classification of maturity of the animals, they did not highlight the number of embryos and the body weights of the pregnant animals. Based on their results, the smallest female, which was classified as mature, was only 1.75 kg, but they did not mention if this animal was pregnant or not. In another scientific work, carried out in Argentina, the number of embryos had been examined through three years and in different months. The minimum number of embryos was 3.80 in June, and the maximum was 9.50 in December, and the mean in total was 6.23 ± 1.45 [42].

This study is among the first that investigates body condition, reproductive performance, and their interactions in nutria in its introduced range in Central Europe. To this end, we collected 69 animals in Slovakia, then executed anatomical postmortem investigations in the laboratory. According to [8], the niche equivalence and similarity tests in invasive nutria showed niches with a moderate-to-high level of overlap in Europe compared to their native habitats. Therefore, we investigated whether the nutria in Central Europe shows a good condition and high reproductive performance in the wild similar to those countries where it lives as a native species [43]. In particular, this study is aimed at responding to the following fundamental questions: (a) Are the individuals in the wild in good physiological condition based on the body sizes of males and females? (b) How rapid is the nutria reproduction rate in this non-native population?

## 2. Materials and Methods

### 2.1. Study Area

**Figure 1:**
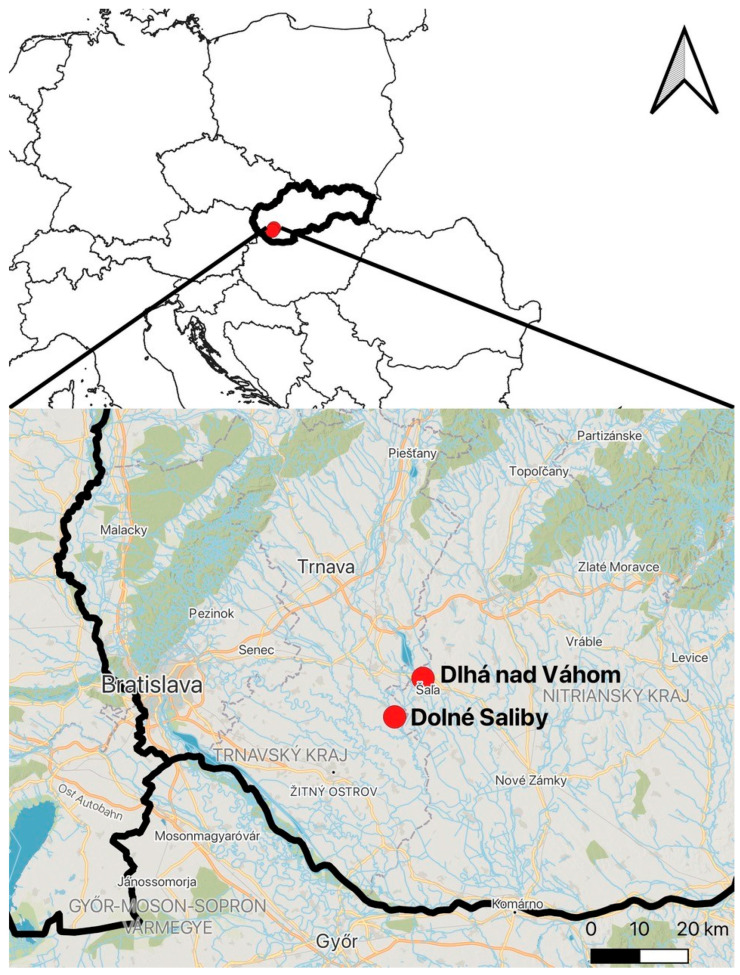
The research areas, the two settlements, Dlhá nad Váhom (Vághosszúfalu) and Dolné Saliby (Alsószeli) in Slovakia. The Hungarian–Slovak border is marked by a black line running along the Danube River, which crosses Bratislava. (Created by Hanna Bijl).

The animals were collected by hunting (i.e., shooting and trapping) in two different hunting clubs in Slovakia. The study areas were located in the southwestern part of the country, approximately 70–80 km away from Bratislava (the capital) and 60–70 km away from the Hungarian state border. The hunting grounds were near Dlhá nad Váhom (Vághosszúfalu) (48°10′20″, 17°51′37″) and Dolné Saliby (Alsószeli) (48°06′, 17°47′) (Figure 1).

The hunting association in Dlhá nad Váhom (Vághosszúfalu) is an organisation for small game management, which manages an 890 ha big hunting area. The nutria, the game species that is the focus of our research, has appeared in this area since the beginning of the 2000s and its population is still growing.

The area is surrounded by one big and two small canals that cross the location of our research, and two smaller-sized fishponds can be found within the area. River Váh (Vág) flows on the border of it. Regarding the topography of the place, it is a flatland mostly covered by cultivated lands with some edges, and there is a small, forested area, as well.

The hunting ground in Dolné Saliby (Alsószeli) is nearly 1900 ha big, with similar habitat characteristics. The nutria has been occurring here since the 1990s.

### 2.2. Field Sample and Data Collection

During the research period between 2022 and 2024, six sample collecting trips, each up to three days, were organised. The animals were harvested mainly by firearms, but additional traps were also set, most by the first author (B.B.) with the help of his local guides. We used different types of live-capture cage traps. Among the selected trap types, a single two-door (sliding door) trap of a size 35 × 45 × 150 cm was used only once, but successfully; a single, newer type of two-door Tomahawk trap measuring 19 × 26 × 102 cm, two times without capture (probably due to its small size); one to four one-door traps (drop door) measuring 25 × 25 × 100 cm in three out of six occasions; and eleven of the newer, foldable version of one-door Tomahawk traps (31 × 31 × 94 cm) during the last trip.

**Figure 2:**
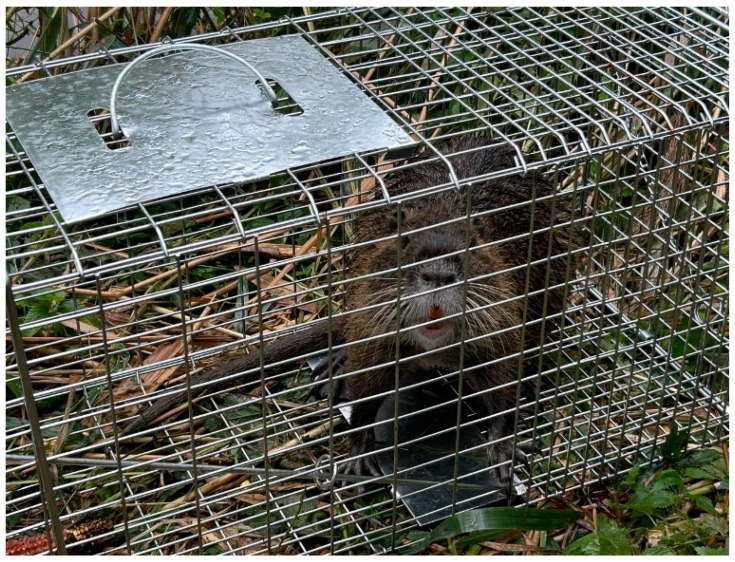
A captured nutria in the foldable cage trap.

Since the traps captured the nutrias alive (Figure 2), the animals were humanly euthanized with a 22 LR firearm in the fastest way to minimise animal suffering. In all cases, the animals’ welfare was the main priority.

Hunting, in general, may cause sampling bias, if individual animals are not captured randomly. To the best of our knowledge, there is no documented evidence of sex- or age-specific behavioural differences in *Myocastor coypus* that would systematically bias our hunting-based sampling method. To address this issue, the location of the traps and hunting trips were selected so that it randomly spanned over the study area.

This means the individuals were not selected based on sex and age. In addition, the trap sizes were selected so that nutrias of different sizes could be caught. We performed the nutria hunting, trapping, and subsequent euthanasia in full compliance with all Slovakian legislation and EU directives, and with the consent, approval, and support of the competent hunting authority.

For trap setups and hunting, we selected bait sites in the stream banks to attract nutrias and to make it easier to humanely shoot them, but we also stalked along the banks of water bodies. In the latter case, we could check more parts of a given area. Most of the high seats were placed near to the nutria burrows. The traps, in particular, were placed in sites which were baited formerly or were near to the tracks of the animals, or in banks of water bodies, which were found to be suitable for capturing. As bait, we mostly used apple and/or grain or whole maize. During the six research trips, we were able to capture 17 individuals, and 52 were harvested by firearms. As a whole, we were able to collect 69 specimens of nutria. From them, 21 were harvested in Dlhá nad Váhom (Vághosszúfalu), of which 1 was trapped and 20 were shot, and 48 in Dolné Saliby (Alsószeli), of which 15 were trapped and 33 were shot.

### 2.3. Laboratory Analysis

The carcasses were kept in the freezer at −18 °C until laboratory analysis. As the first step, we checked the general status of the individual, like its fur and teeth, and the sex of the animals was determined externally based on the anogenital distance (AGD, for males > for females) and the presence of a penis, which is possible to push out. The length and width values were measured in centimetres (cm) in every case, while the body weight was measured in kilograms (kg), and both the weight of the testicles and embryos in grams (g). To measure the body weight of the animals, we used a spring scale, and a digital scale was used for measuring other mass data.

The body length of the individuals was measured by a measuring tape from the tip of the nose to the base of the tail. Then, we measured the length of the tail, from the base to the tip. The length and the width (zygomatic distance) of the head was measured by a calliper. The head length was measured from the tip of the nose to the tip of the occipital bone. We obtained the value of the head width by measuring the distance between the two widest points of the head. For the measurements we used the external points of the zygomatic bones. The length of the hindleg was measured by tape from the femur head to the tip of the middle finger. The length of the sole was also measured by calliper, and the measurement was performed from the tip of the heel to the tip of the middle finger.

The other important measurements were related to the size of internal organs. In the case of the males, we examined the weight and the extension of the testicles; in the female individuals, we checked the uterus. If an embryo was found, we registered the weight of each embryo in grams (g) by using a digital scale, and then the length of each embryo was reported in centimetres (cm) by calliper. The sexing of an embryo was performed by visual inspection based on the AGD, as explained above. However, the sex of an embryo could only be determined if the embryo was in a late developmental stage.

### 2.4. Data Evaluation

The body condition of the individuals was measured by the Body Mass Index (BMI) [44,45,46] as:

BMI = body weight (kg) divided by the square of the nose-to-base of the tail length (m). The testis volume was expressed from the testis length and width data using the calculation for the volume of an ellipsoid in cm:

V (cm^3^) = π/6 × length × width^2^ (1)

Those data, which have been presented in the cases of both sexes and age classes (e.g., BMI, body weight, body length, etc.), were compared in order to obtain the sex-specific estimates of the size differences.

The four groups did not always follow normal distribution, based on the Shapiro–Wilk test; thus, a series of Kruskal–Wallis tests were performed on body weight, BMI, head length, tail length, and hind foot length. In the case of the body length and head width, however, we performed a one-way ANOVA with Duncan range test.

In a similar way, the proportion of pregnant females and the average number of embryos per female were reported by visual inspection. In the case of the comparison of the spring and autumn periods in terms of fertility (number of offspring) and the testicles’ weight and volume, we performed a series of Mann–Whitney U tests. Samples from March and May were merged into the spring season, and those from September and October into autumn. The effect of BMI on fertility was tested by linear regression.

For the statistical analyses we used the SPSS 29.0.1.0 software.

## 3. Results

### 3.1. Body Characteristics

#### 3.1.1. Phenotypical Characteristics

From the investigated 69 individuals, 44 were males, including 25 juveniles and 19 adults, and 25 were females, with 14 adults and 11 juveniles.

During the research trips, besides the common brownish fur variety, we were also able to harvest nutrias with unusual colouring patterns: one with a blend of grey and silver fur colour, called “silver nutria” by former nutria breeders [16], and a few individuals with lighter and paler coat colour.

Most of the harvested animals had healthy incisor teeth. Only in six cases the incisors were broken to some extent; moreover, in one individual, the size of the incisors was longer than average, and in another individual the incisor colour was different (black and white, not orange).

The biggest nutria among harvested animals was a male individual, weighing 10.1 kg, with the body measured at 70 cm long. While there was also a female with approximately the same body weight, it was a pregnant individual with 10 embryos, and its length was smaller, 56.1 cm.

#### 3.1.2. Statistical Comparisons Between Sexes and Age Groups

The results of the statistical comparisons among the two sexes and two age class groups (Table 1a,b) revealed statistically significant differences for each of the measured body parameters. While the body weight (KW = 47.704, df = 3, *p* < 0.001) was similar between both the adult males and females, and the juvenile males and females, the adults differed from the juveniles according to Dunn’s test (*p* < 0.001).

Similarly, for the body length (F_3,65_ = 45.796, *p* < 0.001), no difference was revealed between the adult males and females, but the juvenile males were longer than the females, and adults differed from the juveniles according to the Duncan range test (*p* < 0.001). The BMI (KW = 28.95, df = 3, *p* < 0.001), the tail length (KW = 14.855, df = 3, *p* = 0.002), and the hindfoot length (KW = 39.637, df = 3, *p* < 0.001), just like the body weight, did not show differences between sexes, but did show differences between the age classes in all cases (*p* < 0.05).

The head length (KW = 46.902, df = 3, *p* < 0.001) and head width (F_3,62_ = 31.302, *p* < 0.001) were similar between juvenile males and females, but they differed significantly among adult males and females, and among adults and juveniles (*p* < 0.05).

### 3.2. Reproductive Characteristics

#### 3.2.1. Males

**Figure 3:**
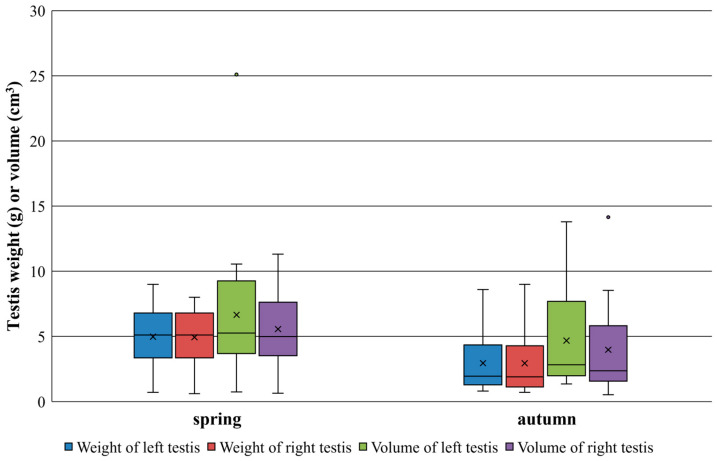
A comparison of testicles’ weight and volume in nutrias between spring and autumn. The dots are outliers (extreme values).

The testis weight and volume were significantly higher in spring than autumn (left testis weight: U = 317.5, n_1_ = 17, n_2_ = 24, *p* = 0.005; right testis weight: U = 314.5, n_1_ = 17, n_2_ = 24, *p* = 0.006; right testis volume: U = 277.5, n_1_ = 16, n_2_ = 24, *p* = 0.035), except for the left testis volume (U = 278, n_1_ = 17, n_2_ = 24, *p* = 0.076) (Figure 3).

#### 3.2.2. Females

**Figure 4:**
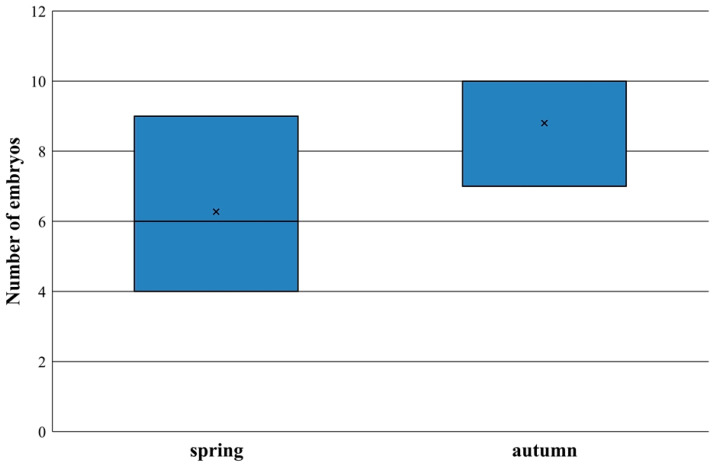
The median number of embryos found in the female nutrias during the spring and autumn months. The × shows the mean value.

Following visual examinations of the uterus, we found that among the 25 female specimens, 16 (64%) were pregnant, including 3 with body sizes characteristic of young nutrias. Among the pregnant female nutrias, 9 of 10 (90%) individuals harvested in spring were pregnant and 7 of 15 (47%) individuals were pregnant in autumn. The total number of embryos found was 113 and the average number of embryos per female was 6.94 ± 2.22. The average number of the embryos per female in spring was 6.27 ± 2.05 and, in autumn, the average number was 8.8 ± 1.64, with no statistically significant difference (U = 18.5, n_1_ = 9, n_2_ = 7, *p* = 0.174) (Figure 4). However, the embryos were less developed in spring than in autumn (average weight: 22.40 ± 41.61 vs. 74.42 ± 118.88).

No clear relationship between the BMI and the fertility of females, i.e., the number of embryos was found (R^2^ = 0.017, F_1,14_ = 0.243, *p* = 0.629). Based on the results, it seems that the condition of the pregnant specimens does not influence the number and the developmental stage of the embryos, so the nutria is able to successfully reproduce even in worse conditional status.

## 4. Discussion

The current study constitutes the first postmortem laboratory analyses of invasive nutrias’ body condition and reproductive potential reported from a free-living population in the Central Europe region. Our results can help in elaborating a more effective management plan to contain the spread of this species across Central Europe

In the current study, we found statistically significant age class differences in the body sizes. However, no sexual dimorphism was observed in the study populations. Similar patterns have been reported from studies on a captive nutria population in Poland [47] and a wild population in Italy [48]. Our results show that the juvenile-to-adult ratio of the estimated body length parameters is around 0.7–0.8; meanwhile, for body weight, this ratio is only 0.5. It reflects that the juveniles can reach the maximum size of their body and body parts earlier, and the adult body weight accumulation is comparatively slower. In a study on a farm-bred nutria population in Czechia, sexual dimorphism in growth was observed in individuals as early as three months of age. The live weight of males increased by approximately 13% each month between six and eight months, whereas the live weight of females increased by approximately 6%. The growth intensity of nutrias decreased with age, mainly from six months [49]. In our study, however, the average body size of nutria reached 7 kg of weight and 60 cm of length for adults, which could suggest that only a few individuals can have a significant local impact on their environment by digging large burrow systems and consuming large amount of plant biomass [18].

Guichón et al. [43] reported that, in an indigenous coypu population in Argentina, nutrias develop to a smaller maximum body size (approximately 20–30% less) than introduced populations in the western parts of Europe such as in the UK and in France. The same pattern was observed in the studied population in Central Europe. Early maturity and comparative larger body size of invasive European populations compared to indigenous coypus could represent an evolutionary response to lower winter temperatures in Europe, which results in the higher mortality rates. A larger body is beneficial in more efficient thermoregulation through a substantial increase in fat reserves.

Investigation of male individuals’ reproduction suggests that the reproduction can be restricted to a subset of adult individuals and to spring months, since adults showed much bigger testis sizes than juvenile males, and also their testes were significantly bigger in the spring than in the autumn period. Nevertheless, considering the 120–125-day-long gestation period [50], the reproduction should have happened in early November for the pregnant nutrias caught in early March.

Female nutrias reproduce throughout the year, and we found pregnant individuals both in spring and autumn (nine vs. seven). Similarly to our results, Courtalon et al. [42] also detected two peaks in births, one in spring and another one in mid-autumn in native situations in Argentina.

Our data suggest that nutrias seem to reproduce comparatively faster in the wild in the Central European environment, because from 25 harvested females, 16 (64%) were pregnant. Courtalon et al. [42] measured a median annual pregnancy rate of 0.45 and 0.88 for two consecutive years in a native Argentine population. However, Cocchi and Riga [48] published the observation during an eradication programme in Italy, where pregnant females constantly represented a high percentage of the mature females (monthly percentage higher than 60%).

Based on Russian research on the reproduction of the studied species, the female nutrias come to oestrus every 25–30 days, i.e., the average of the sexual cycle’s duration is 27.15 ± 7.36 days [28]. We have similar observations supporting this statement during our study. In one incident, a female individual was captured in a trap alongside her young cub. A closer examination of the female specimen in the laboratory, revealed four well-developed embryos inside its uterus, meaning that the female nutria had still been raising an offspring while she was already pregnant for the second time.

The litter size in this Slovakian population was higher than that found in Italy (6.94 embryos vs. 5.06–5.52 on average) [48]. Courtalon et al. [42] found a similar median annual litter size of seven cubs for a native population in South America; however, the litter size in the previous year was approximately five cubs. Moreover, Němeček and Tumová [51] measured smaller litter sizes ranging from 3.9 to 5.1 in a Czech breeding station depending on the original breed of three colour morphs.

We found no correlation between body condition and litter size in the Slovakian population, pointing to the fact that nutria can reproduce even under suboptimal conditions. This result is consistent with an earlier study in Italy that did not report any significant sex differences in the condition of mature individuals [48]. Moreover, they calculated that in the Italian population, the condition index averaged 29.73 ± 4.23. Transforming our data using that same condition index [52], the mature males in Slovakia obtained a value of 35.58, while mature females obtained 37.93, which are higher than reported from nutria in Italy. However, the use of BMI, while it is applied in related species, may not be the optimal indicator of body condition for *Myocastor coypus* without further validation.

In some rodents, a strong relationship can be found between body condition and reproduction [33,34], meaning the better the body condition, the better the fecundity. However, sometimes food availability is more important [35] for reproduction than body condition. Based on our data, body condition has no effect on the reproductive performance of the nutria in Slovakia, most likely due to an abundant food supply throughout the year. Food abundance could result in a higher reproductive output and recruitment rate in this population, since all the different-body-size females could be fertilised, successfully giving birth to new offsprings.

It is noteworthy that studies that use hunted or trapped individuals are prone to potential hunting bias that can cause a smaller sampling size of the females and may decrease the power of the statistical tests between the body parameters of the sexes. Moreover, it can also be a limit in the precise estimation of the reproductive performance of the females since not all female variants in the populations have been represented by the subset of hunted or trapped individuals. Nevertheless, such studies can still provide the necessary first-step level of information that is required to establish an efficient ecosystem management plan to contain the spread of invasive species and minimise their negative impacts on local ecosystems.

## 5. Conclusions

Based on our research, we support our hypothesis that nutria can survive and reproduce effectively in the Central European wild habitats. Our estimates of body condition parameters were comparable with values available from the native range of the species as well as from other European countries, where nutria have already been well-established. Slovakia is connected to its neighbouring countries by water systems, providing great opportunities for nutria to establish new founder populations at the cross-border areas, e.g., with Hungary. In the case of the neighbouring Hungary, the species is not hunted; its legal status and management plans are different. It is important to be prepared for the eradication of the appearing individuals in new areas by establishing the adequate legal status for the species and identifying the most effective interventions. Since the female nutria is able to breed during the whole year, we suggest the eradication of the species needs to be planned all year round. However, based on our results, the number of pregnant females is higher in spring, which means the hunting pressure should be stronger in late winter or early spring. Moreover, public awareness is also crucial in order to introduce invasive nutria to the public and to highlight the problems that they can cause.

## Figures and Tables

**Table 1:** (**a**) The body weight, length, and Body Mass Index, (**b**) tail, hind foot, head length and head width aimed at different sex and age categories of the nutria. The mean, standard deviation (SD) and the maximum and minimum values are provided for each parameter. Statistical differences revealed among groups are shown in the Difference (Diff.) column by different uppercase letters at the significance level signed in the *p* value column.

**(a) Measurement**	**Sex and Age**	**Mean**	**SD**	**Min.**	**Max.**	**Diff.**	***p *Value**
Body weight	Adult male	6.74	1.33	4.5	10.1	A	<0.001
	Adult female	6.69	1.36	5	10.1	A	
	Juvenile male	3.36	1.16	1.9	7.1	B	
	Juvenile female	2.74	1.2	1.3	4.6	B	
Body length	Adult male	57.43	5.17	49	70	A	<0.001
	Adult female	56.29	2.90	50.5	61	A	
	Juvenile male	44.11	5.57	35	58	B	
	Juvenile female	40.15	5.77	30.5	48.1	C	
Body Mass Index	Adult male	20.28	1.65	16.94	23.81	A	<0.001
	Adult female	21.21	4.7	15.39	32.09	A	
	Juvenile male	16.77	2.14	11.30	21.11	B	
	Juvenile female	16.03	2.91	12.31	20.3	B	
**(b) Measurement**	**Sex and Age**	**Mean**	**SD**	**Min.**	**Max.**	**Diff.**	***p *Value **
Tail length	Adult male	38.82	7.97	13.6	49.6	A	=0.002
	Adult female	39.27	3.91	31.5	43.8	A	
	Juvenile male	34.99	4.40	25.2	43.2	B	
	Juvenile female	32.57	6.53	22.7	43.5	B	
Head length	Adult male	13.4	1.49	11.7	18.6	A	<0.001
	Adult female	11.71	0.7	10.55	12.8	B	
	Juvenile male	9.99	1.32	7.8	12.94	C	
	Juvenile female	9.07	1.34	6.8	11.2	C	
Head width	Adult male	8.44	0.75	7.1	10.05	A	<0.001
	Adult female	7.66	0.56	6.7	8.6	B	
	Juvenile male	6.48	0.86	5	8.76	C	
	Juvenile female	5.97	0.91	4.7	7.2	C	
Hindfoot length	Adult male	13.69	3.26	11.5	26.8	A	<0.001
	Adult female	12.52	0.79	11.8	14.5	A	
	Juvenile male	11.24	0.71	9.3	12.5	B	
	Juvenile female	10.48	1.56	8.2	13.1	B	

## Data Availability

Data can be obtained from the authors upon reasonable request.

## Abbreviations

The following abbreviations are used in this manuscript:

AGD	Anogenital Distance
BMI	Body Mass Index
KW	Kruskal–Wallis
SD	Standard Deviation
